# Hashimoto’s Thyroiditis Is Associated With Central Lymph Node Metastasis in Classical Papillary Thyroid Cancer: Analysis from a High-Volume Single-Center Experience

**DOI:** 10.3389/fendo.2022.868606

**Published:** 2022-05-20

**Authors:** Bin Zeng, Yu Min, Yang Feng, Ke Xiang, Hang Chen, Zijing Lin

**Affiliations:** Department of Breast and Thyroid Surgery, The Second Affiliated Hospital of Chongqing Medical University, Chongqing, China

**Keywords:** papillary thyroid carcinoma, Hashimoto’s thyroiditis, central lymph node metastasis, nomogram, risk factor

## Abstract

**Purpose:**

Central lymph node metastasis (CLNM) is regarded as a predictor for local recurrence in patients with papillary thyroid carcinoma (PTC) but the role of prophylactic central lymph node dissection (CLND) is controversial. Our study aims to identify the clinical factors associated with CLNM and develop a nomogram for making individualized clinical decisions.

**Method:**

The perioperative data of 1,054 consecutive patients between Jan 2019 and April 2021, in our center, were reviewed and analyzed. A total of 747 patients with histopathologically confirmed classical PTC were included as the training cohort and 374 (50% training cases) patients were randomly selected to build a validating cohort *via* internal bootstrap analysis. Univariate and multivariate logistic regression were used to analyze the correlation between clinicopathological characteristics and CLNM.

**Result:**

In the training cohort, 33.6% (251/747) of patients with classical PTC were confirmed with CLNM. And the CLNM was determined in 31.4% (168/535) of non-Hashimoto’s thyroiditis (HT) patients versus 39.2% (83/212) in HT patients (p=0.043). Four factors including gender, age, size, and HT status were confirmed significantly associated with CLNM. The established nomogram showed good discrimination and consistency with a C-index of 0.703, supported by the internal validation cohort with a C-index of 0.701. The decision curve analysis showed the nomogram has promising clinical feasibility.

**Conclusion:**

Our study suggested that classical PTC patients with features like male gender, age<55 years old, tumor size>1cm, and HT condition had a higher risk of CLNM. And the nomogram we developed can help surgeons make individualized clinical decisions in classical PTC patients during preoperative and intraoperative management.

## Introduction

Over the past few years, the incidence rate of papillary thyroid carcinoma (PTC) has rapidly increased on a global scale, which has become the most prevalent endocrine-related malignancy (1–3). Despite a favorable long-term prognosis (4, 5), a minority of patients with central lymph node metastasis (CLNM) and even lateral lymph node metastasis (LLNM) showed higher recurrent risk and impaired disease-free survival (6–8). For instance, in one large-scale retrospective study from Italy, Sapuppo et al. determined that PTC patients with N1b had a higher risk of suffering distant metastases than N1a and N0 patients (7). Moreover, PTC patients with high-volume CLNM had a higher risk of local recurrence (7).

Currently, great achievements have been made in a range of works to determine the risk factors in the lymph node metastasis of PTC (9–11). Many clinical characteristics including but not limited to the male, younger age, larger tumor size, multifocal lesions, and extracapsular spread were regarded as the independent risk factors involving the lymph node metastasis of PTC.

Hashimoto’s thyroiditis (HT), also known as chronic lymphocytic thyroiditis, is the most prevalent autoimmune disease nowadays (12). It causes chronic inflammation and damage in thyroid tissues *via* over-expression of thyroid globulin antibody (TgAb) and thyroid peroxidase antibody (TPOAb), leading to the condition of hypothyroidism in about 20-30% of patients, ultimately. Although a high concurrent rate of HT and PTC has been observed in the last century (13, 14), the relationship between these two diseases remains highly controversial. Some studies suggested that PTC patients who coexisted with HT had lower staging and better prognosis, compared with non-HT patients (15–19). The potential mechanisms are partly due to the modulation of the tumor microenvironment, the induction of abnormally immune responses and lymphocytic infiltrates in tumor tissue. However, some other studies reported that the coexistence of HT had no protective effect on patient outcomes (20, 21). Furthermore, some scholars even suggested that high thyroid antibody status, especially serum TgAb level, could be a risk factor in promoting the CLNM of PTC patients (22, 23). More clinical trials focusing on the effect of HT on the progression of PTC are merited.

The purpose of this retrospective study was to evaluate the role of clinicopathological factors including HT condition in predicting the CLNM for PTC patients. Also, we aimed to establish a prediction model based on the extracted clinicopathological factors to help surgeons preoperatively make individualized clinical decisions in terms of whether the prophylactic central lymph node dissection (CLND) is warranted.

## Material and Method

### Data Source

Patients with the postoperative histopathological diagnosis of classical PTC at the Second Affiliated Hospital of Chongqing Medical University between January 2019 and April 2021 were reviewed and analyzed.

**Figure 1:**
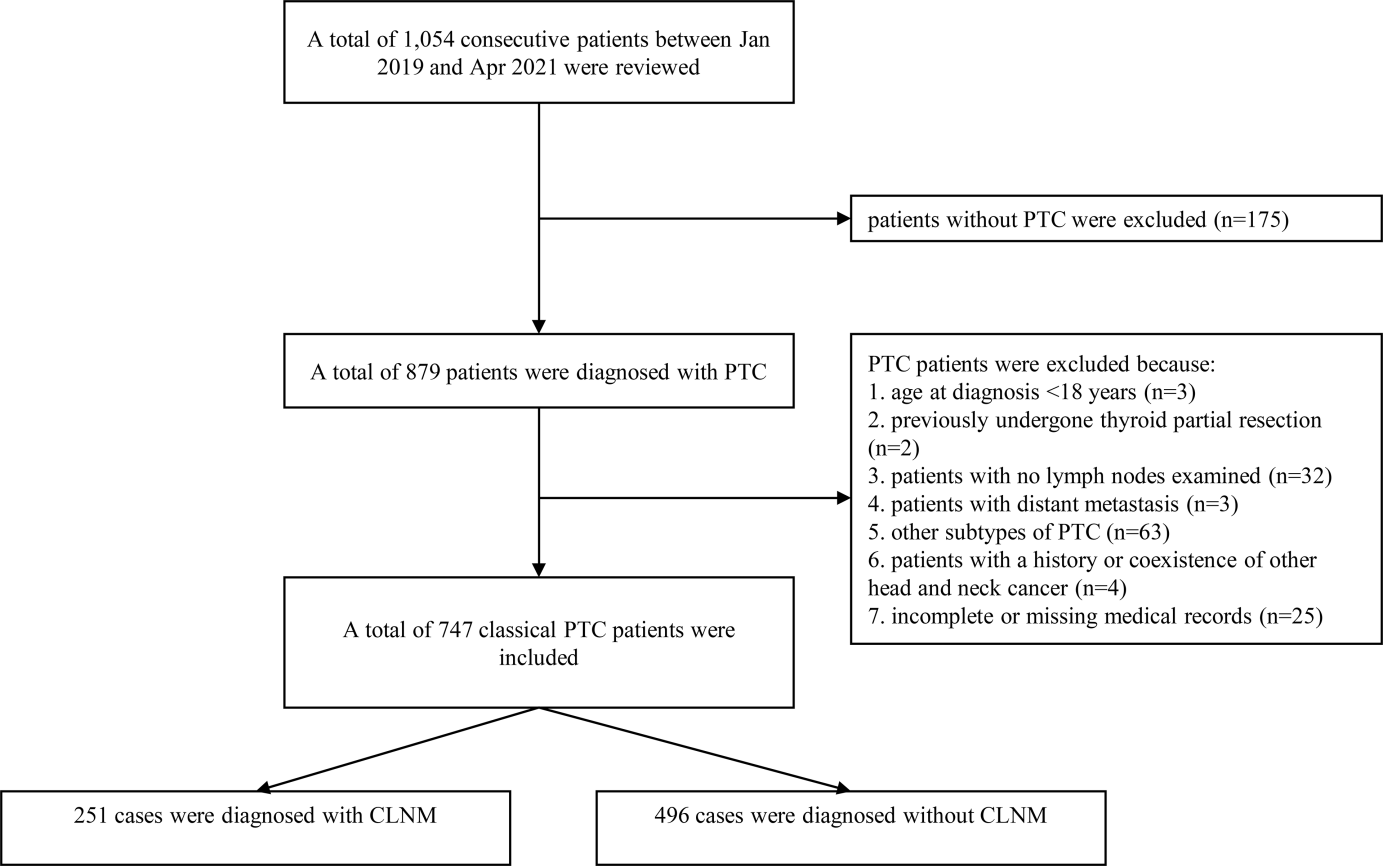
The patients’ selecting process in our department.

The inclusion and exclusion criteria among the training cohort were as follows: patients were enrolled during the study period using the following inclusion criteria: 1) aged between 18-75 years, and 2) underwent thyroidectomy and central lymph node exams. Patients were excluded during the study period using the following exclusion criteria: 1) no histologically proven PTC, 2) no lymph nodes found in the final pathological report, 3) other subtypes of thyroid cancer, 4) history or coexistence of other head and neck cancer, and 5) incomplete or missing medical records (**Figure 1**).

### Surgical Extension

All of the surgical procedures (thyroidectomy and CLND) in our department were conducted by Prof. Yin, Prof. Ming, and Prof. Luo who are the senior specialists in general surgery, especially in terms of thyroid and breast surgery. The surgical extension of thyroid surgery followed the Chinese guideline (24). Specifically, central lymph node dissection is generally recommended, whereas lateral neck compartmental lymph nodes should be reasonably and selectively dissected. For tumors with a diameter> 4cm, or a naked thyroid infringement (clinical T4), or thyroid cancer patients with clinically significant local lymph node metastasis (clinical N1), first-proceeds should be total thyroidectomy. For the thyroid cancer patient with cN0 with a tumor diameter <4 cm, the treatment should be lobectomy, unless a clear indicator for resection of the contralateral gland, and the frozen biopsy was routinely performed in our department. Prophylactic or therapeutic unilateral central lymph node dissection was routinely performed in our department, whereas bilateral central lymph node dissection should be reasonably and selectively dissected (unless cN1 of contralateral central lymph node or multifocal lesions).

### Variable Definition and Classification

The following information was collected to establish a retrospective database: gender, age, tumor size, extrathyroidal invasion, the status of CLNM and LLNM, HT status. HT was diagnosed based on one of the following criteria: i) the ultrasound examination revealed diffuse enlargement of thyroid with abundant blood flow combined with TPOAb > 34 IU/L or TgAb > 115 IU/L; ii) postoperative pathology confirmed diffusion lymphocytic thyroiditis), multifocality (more than two primary tumor focus) and BRAF^V600E^ mutation testing results. All acquired surgical specimens were examined by the pathologists from the department of pathology of the Second Affiliated Hospital of Chongqing Medical University.

### Statistical Analysis

The baseline characteristics by HT were compared using Chi-Square tests. Univariate and multivariate logistic regression analyses were used to identify the independent risk factors in patients. A two-tailed p-value of <0.05 was defined as the criterion for variable deletion when performing multivariate analyses. A nomogram for predicting the CLNM based on the results of the multivariate logistic regression analysis was developed and evaluated by a decision curve analysis (DCA) curve. All analyses were performed using the “SPSS version 24.0” and “R version 4.0.2” software.

## Results

### Baseline Clinicopathological Characteristics

A total of 747 (157 men; 590 women) classical PTC patients were enrolled in the training cohort and 374 patients were included as the validating cohort for developing and internally validating the clinical risk-stratifying model (**Table 1**). There were no significantly different clinicopathological characteristics observed in the two cohorts. However, a remarkable difference was determined in the clinical features of these patients when they were stratified by HT status (**Table 2**). Among these patients, female patients (590/747, 79%) accounted for most of the study group, compared with male patients (157/747, 21%). Additionally, the CLNM has ultimately been confirmed in 251 (33.6%) PTC patients, and 58 (7.8%) of patients were histopathologically confirmed with LLNM. Collectively, 212 (28.4%) PTC patients were diagnosed with coexisted with HT, and a significantly higher proportion (94.3%) of the female population was found in patients with concurrent HT (*p*<0.001). Besides, the CLNM was determined in 168 (31.4%) non-HT patients and 83 (39.2%) HT patients. However, there was no significant difference between these two datasets in terms of the prevalence of LLNM (*p*=0.889). Furthermore, there was no significant difference in age, tumor size, multifocality, extrathyroidal invasion, TNM stage, and BRAF^v600E^ mutation.

**Table 1:** The clinicopathological characteristics of classical PTC patients.

Variables	Subgroup	No. of patients		*P*
		Training cohort(n=747)	Validating cohort(n=374)	
**Age (year)**	<55	589	294	^a^0.938
	≥55	158	80	
**Gender**	male	157	76	^a^0.815
	female	590	298	
**Race**	Chinese	747	374	–
**8^th^ TNM Stage**	I	702	356	^b^0.724
	II	40	16	
	III	5	2	
**Multifocality**	No	466	217	^a^0.173
	Yes	281	157	
**Extrathyroidal invasion**	No	685	344	^a^0.909
	Yes	62	30	
**Primary surgical extension**	TL+CLND	222	117	^a^0.182
	subTT+CLND	46	33	
	TT+CLND	479	224	
**Tumor size (cm)**	≤1	496	244	^b^0.924
	>1 and ≤2	189	95	
	>2 and ≤4	55	31	
	>4	7	4	
**BRAF^V600E^ mutation**	No	45	26	^a^0.749
	Yes	587	287	
	N/A	115	61	
**Clinical lymph node status**	cN0	627	308	^a^0.551
	cN1	120	66	
**HT**	No	535	249	^a^0.085
	Yes	212	125	
**TSH**	/	^*^ 2.71 ± 5.26	2.42 ± 1.61	^c^0.292
**fT3**	/	4.85 ± 1.06	4.84 ± 0.86	^c^0.817
**fT4**	/	^*^ 16.78 ± 3.05	16.85 ± 2.95	^c^0.724
**tT3**	/	^*^ 2.01 ± 5.22	1.81 ± 1.05	^c^0.514
**tT4**	/	^*^ 99.94 ± 21.02	102.14 ± 22.36	^c^0.146
**TgAb**	/	^*^ 184.02 ± 496.64	152.79 ± 350.39	^c^0.282
**TPOAb**	/	^*^ 66.93 ± 119.85	73.41 ± 125.62	^c^0.403
**TG**	/	^*^ 38.31 ± 88.79	44.21 ± 104.02	^c^0.344
**Central LN examined**	/	^*^ 4.20 ± 4.50	4.47 ± 4.93	^c^0.347
**Central LN positive**	/	^*^ 1.12 ± 2.19	1.32 ± 2.51	^c^0.175
**Lateral LN examined**	/	^*^ 1.21 ± 4.46	1.40 ± 4.85	^c^0.515
**Lateral LN positive**	/	^*^ 0.30 ± 1.43	0.33 ± 1.54	^c^0.723
**Central LNM**	No	496	239	^a^0.424
	Yes	251	135	
**Lateral LNM**	No	689	341	^a^0.563
	Yes	58	33	

*: Mean ± HT, Hashimoto’s thyroiditis; PTC, papillary thyroid carcinoma; cN0, clinically lymph node-negative; cN1, clinically lymph node-positive; TL, thyroid lobectomy; CLND, central lymph node dissection; subTT, subtotal thyroidectomy; TT, total thyroidectomy; LNM, lymph node metastasis; LN, lymph node. ^a^Pearson’s Chi-squared test ^b^Two-tail Fisher exact test ^c^Student’s two-tail t-test.

**Table 2:** Clinicopathological features of 747 PTC patients with HT or without HT in the training cohort.

Variables	Subgroup	No. (%) of patients		*P*
		without HT (n=535)	with HT (n=212)	
**Gender**	male	145 (27.1)	12 (5.7)	**<0.001**
	female	390 (72.9)	200 (94.3)	
**Age**	<55	414 (77.4)	175 (82.5)	0.119
	≥55	121 (22.6)	37 (17.5)	
**Tumor size (cm)**	≤1	361 (67.5)	135 (63.7)	0.175
	>1 and ≤2	127 (23.7)	62 (29.2)	
	>2 and ≤4	40 (7.5)	15 (7.1)	
	>4	7 (1.3)	0 (0.0)	
**Multifocality**	positive	202 (37.8)	79 (37.3)	0.900
	negative	333 (62.2)	133 (62.7)	
**Extrathyroidal invasion**	positive	46 (8.6)	16 (7.5)	0.639
	negative	489 (91.4)	196 (92.5)	
**BRAF mutation**	No	30 (5.6)	15 (7.1)	0.719
	Yes	421 (78.7)	166 (78.3)	
	NA	84 (15.7)	31 (14.6)	
**TSH**	/	^*^2.54 ± 5.190	3.14 ± 5.436	^¶^0.158
**fT3**	/	^*^4.89 ± 1.075	4.75 ± 1.001	^¶^0.314
**fT4**	/	^*^16.74 ± 2.933	16.87 ± 3.321	^¶^0.348
**tT3**	/	^*^2.12 ± 6.128	1.69 ± 0.361	^¶^0.937
**tT4**	/	^*^100.10 ± 20.773	99.53 ± 21.728	^¶^0.162
**TgAb**	/	^*^64.21 ± 289.082	481.89 ± 727.597	**<0.0001** ^¶^
**TPOAb**	/	^*^29.58 ± 57.723	160.40 ± 172.732	**<0.0001** ^¶^
**TG**	/	^*^41.27 ± 89.908	31.05 ± 85.510	^¶^0.494
**Central LN examined**	/	^*^3.30 ± 3.739	6.46 ± 5.390	**<0.0001** ^¶^
**Central LN positive**	/	^*^1.03 ± 2.120	1.33 ± 2.372	^¶^0.092
**Lateral LN examined**	/	^*^1.17 ± 4.555	1.31 ± 4.242	^¶^0.699
**Lateral LN positive**	/	^*^0.31 ± 1.555	0.25 ± 1.097	^¶^0.584
**Central LNM**	No	367 (68.6)	129 (60.8)	**0.043**
	Yes	168 (31.4)	83 (39.2)	
**Lateral LNM**	No	493 (92.1)	196 (92.5)	0.889
	Yes	42 (7.9)	16 (7.5)	
**TNM stage**	I	504 (94.2)	198 (93.4)	0.462
	II	19 (3.6)	11 (5.2)	
	III	12 (2.2)	3 (1.4)	

*: Mean ± SD; ¶: two-tailed t-test; HT, Hashimoto’s thyroiditis; LNM, lymph node metastasis; NA, not mentioned. Bold values indicate statistical significance (p < 0.05).

### Univariate and Multivariate Analysis of CLNM

We found that male gender (p<0.001), age<55 years old (p=0.014), tumor size>1cm (p<0.001), and presence of HT condition (p=0.044) were the potential risk factors associated with CLNM in PTC patients (**Table 3**). The multivariate analysis confirmed that male gender (OR=2.426, 95%CI: 1.628-3.614, p<0.001), tumor size (1cm<largest diameter ≤2cm, OR=3.315, 95%CI: 2.309-4.760; 2cm< largest diameter ≤4cm, OR=5.270, 95%CI: 2.908- 9.552; largest diameter >4cm, OR=5.072, 95%CI: 1.083-23.748; p<0.001), and HT condition (OR=1.678, 95%CI: 1.161-2.424, p=0.006) were defined as independent risk factors for CLNM. On the contrary, age ≥55 years old (OR=0.583, 95%CI: 0.382-0.890, p<0.001) was a protective factor for CLNM. Regarding to the BRAF^V600E^ status, there was no significant relationship observed in BRAF^V600E^ mutation and risk of CLNM in classical PTC patients (OR=1.162, 95%CI: 0.604-2.234, p=0.666).

**Table 3:** Univariate and multivariate analysis of 747 PTC patients for CLNM.

Variables	Subgroup	Univariable		Multivariable	
		Hazard ratio	*P*	Hazard ratio	*P*
**Gender**	female	Reference	**<0.001**	Reference	**<0.001**
	male	1.945 (1.357, 2.787)		2.426 (1.628, 3.614)	
**Age**	<55	Reference	**0.014**	Reference	**0.012**
	≥55	0.607 (0.409, 0.902)		0.583 (0.382, 0.890)	
**Tumor size (cm)**	≤1	Reference	**<0.001**	Reference	**<0.001**
	>1 and ≤2	3.202 (2.252, 4.552)		3.315 (2.309, 4.760)	
	>2 and ≤4	4.752 (2.667, 8.466)		5.270 (2.908, 9.552)	
	>4	4.224 (0.932, 19.142)		5.072 (1.083, 23.748)	
**Multifocality**	negative	Reference	0.372	/	
	positive	1.153 (0.844, 1.574)			
**BRAF^V600E^ mutation**	No	Reference	0.666	/	
	Yes	1.162 (0.604, 2.234)			
	NA	0.969 (0.460, 2.042)			
**HT**	No	Reference	**0.044**	Reference	**0.006**
	Yes	1.406 (1.010, 1.957)		1.678 (1.161, 2.424)	

CLNM, central lymph node metastasis; HT, Hashimoto’s thyroiditis; NA, not mentioned. Bold values indicate statistical significance (p < 0.05).

### Nomogram Construction and Validation for Predicting CLNM in PTC Patients

**Figure 2:**
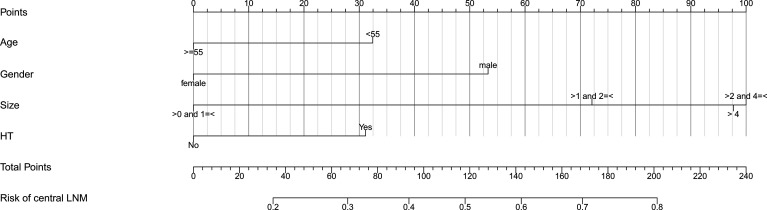
Clinical factor-based nomogram used for predicting the CLNM in PTC patients. CLNM, central lymph node metastasis, HT, Hashimoto’s thyroiditis.

**Figure 3:**
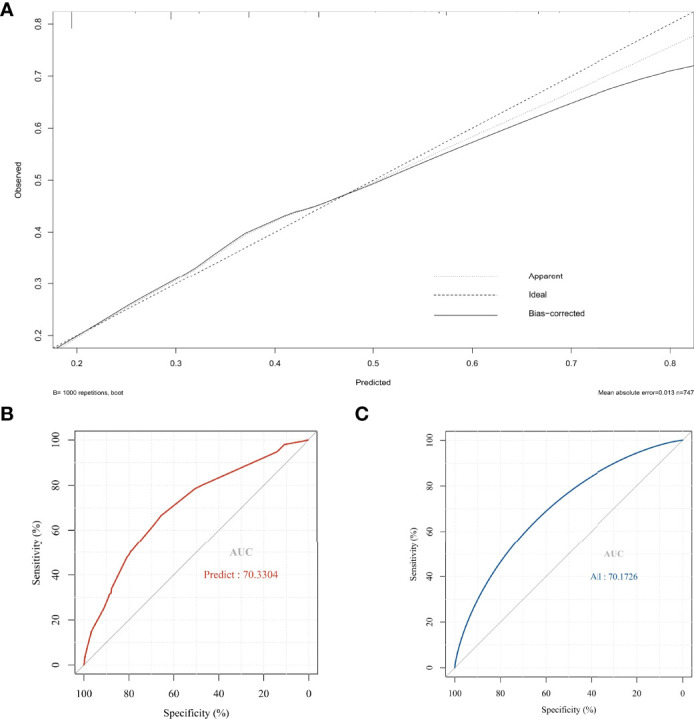
**(A)** The calibration curve for evaluating the accuracy of the nomogram; **(B)** The receiver operating characteristics (ROC) curve and area under the ROC curve (AUC) in the training cohort; **(C)** The receiver operating characteristics (ROC) curve and area under the ROC curve (AUC) in the validating cohort.

**Figure 4:**
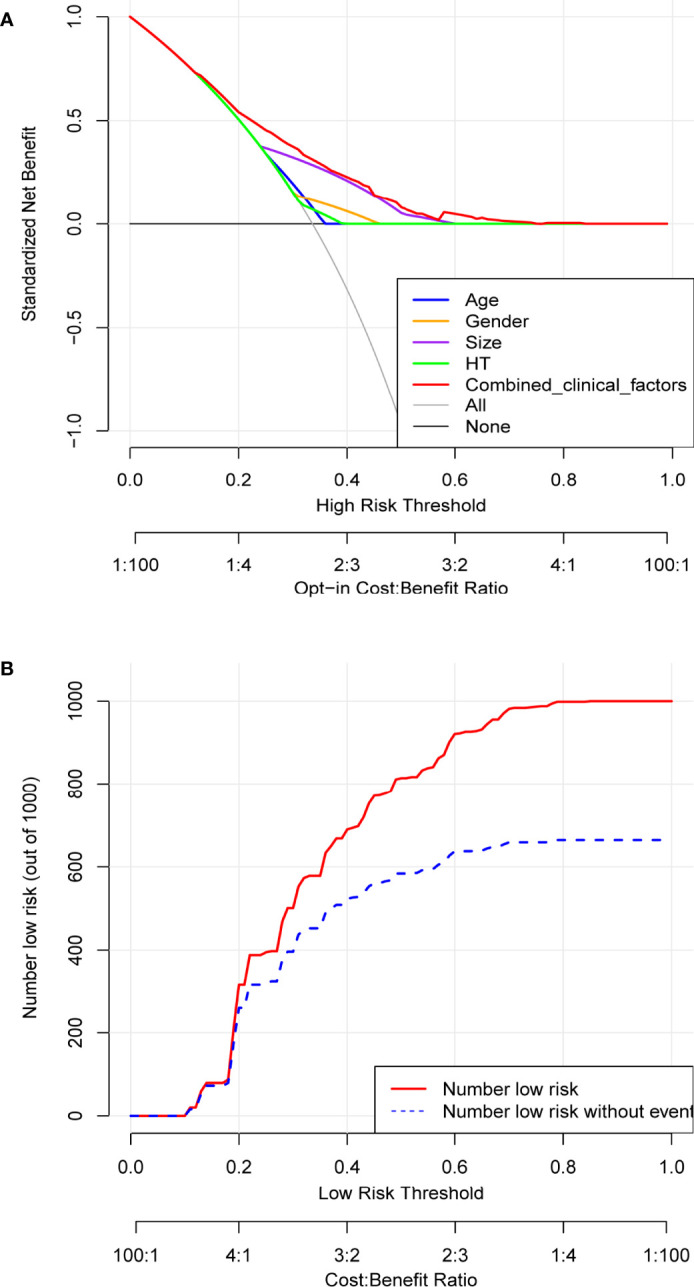
Determination of decision points *via* Decision Curve Analysis (DCA) and Clinical Impact Curve (CIC). **(A)** DCA for the prediction model (EI-Score). The decision curve analysis graphically shows the clinical usefulness of the EI-Score based on a continuum of potential thresholds for central lymph node metastasis (CLNM) (x-axis) and the net benefit of using the EI-Score to stratify patients (y-axis). Net benefit curves are plotted across probability thresholds for 7 options: ‘‘treat all’ assume all patients have CLNM, ‘treat none’ assume no patients have CLNM, treat according to combined clinical factors (age, gender, tumor size, HT), Age, Gender, size, HT and EI-Score. Net benefit = (true positives/N)- (false positives/N) *(weighting factor). Weighting factor = Threshold probability/(1-threshold probability). **(B)** CIC for EI-Score. The red line shows the total number who would be deemed as low risk of CLNM for each risk threshold. The blue line shows how many of those would be true positive (without CLNM).

Based on the independent risk factors determined by multivariate analysis, a nomogram (**Figure 2**) was established for predicting the risk of CLNM in patients with PTC. Patients with the risk factors including gender, age, tumor size, and HT were enrolled in our nomogram model (the score of each factor is shown in **Table 4**) to predict the presence of CLNM in PTC patients. A C-index of 0.703 was achieved in the present model. Moreover, the calibration curve for evaluating the accuracy of the nomogram showed a good internal consistency (**Figure 3A**). Besides, the AUC of the training cohort was 0.703, which was in accordance with the C-index (**Figure 3B**). In addition, an internal cohort with half the study population was developed via random bootstrap analysis, which was used to validate the feasibility of the nomogram and reached an AUC of 0.701 (**Figure 3C**). Furthermore, a decision curve analysis (DCA) was performed to compare the predictive ability between the combined clinical factors nomogram and the single-factor model. The standardized net benefits of the models were comparable and there was a significant overlap between these models. Collectively, the DCA curve showed that the prediction ability of the combined independent risk factors was superior to the single-factor in detecting CLNM for classical PTC patients which would be more effective than a treat-none or treat-all strategy when the threshold probability ranged from 0.2 to 0.8 (**Figure 4A**). To further evaluate the clinical significance of this prediction model, the clinical impact curve (CIC) was delineated (**Figure 4B**). As expected, the CIC results show that among the broad thresholds for CLNM (20–80%), the nomogram was classified as positive and the number of true positives was greater than those of the separated factor model.

**Table 4:** The specific value of clinicopathological features in the nomogram.

Characteristics	Score
**Age**	
≥5 years old	0
<55 years old	32
**Gender**	
female	0
male	53
**Size (cm)**	
>0 and ≤1	0
>1 and ≤2	72
>2 and ≤4	100
>4	98
**Hashimoto’s thyroiditis**	
Not present	0
present	31
**Total point for predicting the CLNM**	
0.2	34
0.3	67
0.4	93
0.5	118
0.6	141
0.7	169
0.8	201

CLNM, central lymph node metastasis.

## Discussion

To date, with an increasing prevalence and overdetection rate of PTC around the world, the management of this disease becomes more precise and particularly crucial. For preventing unnecessary diagnostic workup and possible surgical intervention, two medical societies from East Asia and North America (the Japan Association of Endocrine Surgery and the American Thyroid Association) updated the clinical practice guidelines regarding the indications and strategy for active surveillance (AS) of low-risk PTMC (cT_1_N_0_M_0_) patients, especially for the elderly population (25, 26). It indicated that the treatment modality of differentiated thyroid carcinoma became more conservative in developed countries. Nevertheless, surgical intervention is still the pivotal initial treatment for PTC patients in China (24), especially in terms of patients with suspicious clinically CLNM.

In the present study, we aimed to investigate the risk factors in promoting the CLNM in PTC patients and further establish an individualized model for this subpopulation. The CLNM was histopathologically confirmed in 251 (33.6%) PTC patients which were consistent with other reports (15, 16, 27). Besides, 58 (7.8%) patients were diagnosed to suffer from LLNM which was consistent with Homma’s study (8.4%) (6) but much lower than the observation result (25.6%) made by Yang et al. (27). This divergence might be due to the different study populations and the number of patients. We selected seven variables including gender, age, tumor size, multifocality, BRAF^V600E^ mutation, and HT for univariate analysis. Neither multifocality nor BRAF^V600E^ mutation was associated with CLNM. Due to these differences, approximately 15% of patients did not undergo the fine-needle aspiration (FNA) or BRAF^V600E^ test before the surgery which was a common limitation in recent studies (27, 28). Besides, a relatively smaller sample size compared with other large cohort studies which also contributed to this difference. Hence, gender, age, tumor size, and HT were finally screened out for multivariable analysis, similar to the recently reported studies (29, 30). Interestingly, it is believed that tumor size was one of the pivotal risk factors in CLNM and the risk of CLNM increased as the diameter of the primary nodule increased. However, our data suggested that the highest risk ratio did not appear in tumors with a diameter larger than 4cm, instead of in tumors with a diameter larger than 2cm but smaller than 4cm (**Figure 1**). This phenomenon was potentially associated with the limited sample size (only seven cases) of PTC patients with a diameter larger than 4cm. Reviewing recent guidelines from Asian and western countries (24, 26), the role of prophylactic central lymph node dissection (CLND) in PTC continues to be debatable. For instance, as recommended by the clinical practice guideline derived from the American Thyroid Association, omitting CLND was safe and appropriate for patients with the small primary thyroid tumor, especially in terms of clinically node-negative (cN0) PTC. In addition, one multicenter study determined that there was no significant difference in the risk of regional recurrence among total thyroidectomy (TT) alone, TT with ipsilateral CLND, and TT with bilateral CLND in dealing with cN0 differentiated thyroid carcinoma (DTC) but groups treated with TT alone presented the lowest incidence of postoperative complications (31). Notably, clinical evidence from one meta-analysis (32) report (3,331 cases involved) demonstrated that patients with prophylactic CLND presented a 35% reduction in risk of postoperative locoregional recurrence to those who undergo TT alone during the 5 year follow-up, whereas the overall complication rate including, but limited to, transient hypocalcemia, in the former group was much higher than the latter group. Similarly, a randomized controlled study by Viola et al. (33) also highlighted that there were no clinical advantages in performing prophylactic CLND in patients with PTC with cN0 at neck ultrasound. While the TNM staging system is a common method to predict the disease-specific prognosis of PTC, it could not provide preoperative guidance for surgeons to decide on the precision surgical extension (34). Consequently, a more comprehensive evaluation of risk factors for CLNM in the adult population might bring more clinically significant value for preoperatively clinical decisions in these patients with different risks.

Additionally, in our study, the diagnosis of HT was based on the pathology of the surgical specimen. We observed that there was a lower rate of coexisted HT in male PTC patients than in female PTC patients (7.6% vs. 33.9%, *p*<0.001), which was consistent with previous studies, and no significant difference was found between PTC patients with or without HT except for gender and CLNM. Based on multivariate analysis, the HT condition in our study was determined to be an independent risk factor in CLNM (*p*=0.006), which was partially different from the conclusion in the previous study. Therefore, the role of HT in the progression of PTC is worth discussing.

Currently, a range of works (15, 23, 35, 36), especially retrospective studies, have determined a high concurrent rate of HT and PTC from surgical specimens but the relationship between these two diseases, as well as HT and CLNM, has been controversial. Immunologically, emerging evidence has shown that an abnormal inflammatory response, especially the imbalanced subsets of T cells, NK cells, and cytokines were presented in HT condition (14, 37), which could potentially affect the tumor microenvironment and subsequent prognosis. For instance, *in vitro* experiment, Lubin et al. (38) verified that the presence of background HT contributed to a higher risk of CLNM *via* increased programmed death ligand-1(PD-L1). On the contrary, results from Hu et al. (19) suggested that enhanced MHC class I expression in HT could decrease the PD-L1 and further overcome the CLNM in PTC patients. Serologically, Wen et al. (23) conducted that different thyroid antibody status was significantly associated with the CLNM in PTC patients concurrent with HT. They concluded single TgAb was a risk factor in CLNM, whereas TPOAb played a protective role in preventing CLNM. By contrast, a few studies hold the opposite view on the role of serum TPOAb levels in CLNM (39). The inconsistent results in these studies inspired us to provide our own experience in the present work.

Reviewing similar works on predicting CLNM (15, 27, 28, 40), our study had a partial difference and takes it a step further. Our data indicated that HT was one of the independent risk factors in promoting CLNM which deserved further evaluation. Although the C-index in the previous study achieved 0.764 based on 914 PTC patients (41) and 0.854 based on a sample size of 1,252 PTC patients (27), the C-index of our nomogram was still more than 0.7, indicating that it also has sufficient discrimination ability. The DCA results show that the nomogram we developed has good clinical practical value. Combined with other established nomograms based on ultrasound signatures, our nomogram with clinicopathological characteristics with the strongest risk factors including gender, age, size, and HT can increase the accuracy of predicting CLNM. These prognostic factors collected from preoperative testing could further help surgeons to decide the extent of the initial thyroidectomy and whether prophylactic central neck dissection is warranted.

Nonetheless, the results from the study need to be carefully interpreted and some limitations should be addressed in the following works. First and foremost, the weakness of this cohort is a lack of external validation which limits the clinical application. Thus, the external validation cohorts from multicenter countries are urgently demanded to further evaluate the feasibility of our nomograms. Second, this was a retrospective study from a single-center teaching hospital center which did introduce some selection biases. Moreover, there were only four clinical factors ultimately added to our nomogram, which indicated there might be potential variables waiting to be discovered that could make our nomogram complete and more reliable, including but not limited to body mass index (BMI), preoperative ultrasound signatures, and some laboratory testing results which were previously determined to be associated with CLNM in classical PTC patients (27, 28).

## Conclusion

In summary, several clinical features including male gender, younger age, larger tumor size, and HT status were independent risk factors for CLNM in classical PTC patients. A predicting nomogram based on these clinical risk factors is established to help surgeons make individualized clinical decisions during intraoperative management.

## Data Availability Statement

The original contributions presented in the study are included in the article/supplementary material. Further inquiries can be directed to the corresponding author.

## Ethics Statement

Ethical review and approval was not required for the study on human participants in accordance with the local legislation and institutional requirements. The patients/participants provided their written informed consent to participate in this study.

## Author Contributions

Conception and design: BZ and YM. Administrative support: ZL. Provision of study materials or patients: YF, HC, and YM. Collection and assembly of data: BZ, YM, and KX. Data analysis and interpretation: BZ, YM, and YF. Manuscript writing: All authors. Final approval of manuscript: All authors.

## Conflict of Interest

The authors declare that the research was conducted in the absence of any commercial or financial relationships that could be construed as a potential conflict of interest.

## Publisher’s Note

All claims expressed in this article are solely those of the authors and do not necessarily represent those of their affiliated organizations, or those of the publisher, the editors and the reviewers. Any product that may be evaluated in this article, or claim that may be made by its manufacturer, is not guaranteed or endorsed by the publisher.
